# Tissue Culture—A Sustainable Approach to Explore Plant Stresses

**DOI:** 10.3390/life13030780

**Published:** 2023-03-14

**Authors:** Akila Wijerathna-Yapa, Jayeni Hiti-Bandaralage

**Affiliations:** 1 ARC Centre of Excellence for Plant Success in Nature and Agriculture, The University of Queensland, St Lucia, QLD 4072, Australia; 2 School of Biological Sciences, The University of Queensland, St Lucia, QLD 4072, Australia; 3 Centre for Horticultural Science, Queensland Alliance for Agriculture and Food Innovation, The University of Queensland, Brisbane, QLD 4068, Australia; 4 J&S Plant Biotech, P.O. Box 4700, Sunshine Coast MC, QLD 4560, Australia

**Keywords:** micropropagation, tissue culture, germplasm, molecular breeding, biotechnology

## Abstract

Plants are constantly faced with biotic or abiotic stress, which affects their growth and development. Yield reduction due to biotic and abiotic stresses on economically important crop species causes substantial economic loss at a global level. Breeding for stress tolerance to create elite and superior genotypes has been a common practice for many decades, and plant tissue culture can be an efficient and cost-effective method. Tissue culture is a valuable tool to develop stress tolerance, screen stress tolerance, and elucidate physiological and biochemical changes during stress. In vitro selection carried out under controlled environment conditions in confined spaces is highly effective and cheaper to maintain. This review emphasizes the relevance of plant tissue culture for screening major abiotic stresses, drought, and salinity, and the development of disease resistance. Further emphasis is given to screening metal hyperaccumulators and transgenic technological applications for stress tolerance.

## 1. Introduction

Plant tissue culture satisfies large-scale plant propagation needs and is an essential tool facilitating other biotechnology applications in plant improvement space [1,2,3]. In addition, its importance as a tool and direct application in fundamental studies relating to plant biology, biochemistry, and molecular biology is well recognized. Today, the world population has reached an alarming 8 billion people. Providing food and products, of which the majority is plant-based, has become one of the biggest challenges in front of the human race in this era, while the unprecedented climate change challenges are bigger than ever before [4,5,6]. Various climatic changes as a result of global warming greatly influence agriculture systems around the globe. This includes severe environmental pressures, drought, extreme heat or cold climate, floods, salinity, and exposure to toxic compounds [4]. Human activities in the current production-based economy also contribute toward changes in soil and environmental conditions due to the accumulation of toxins and chemical elutes released to the environment in production processors [7]. The flip side of this is the reduction in agricultural production, flagging food security due to the decrease in plant growth and development due to various environmental stress factors and the decline and scarcity of suitable agricultural land.

The focus on plant stress-related research has gained substantial momentum over the last four decades, especially on the impact of stress factors such as water deficiency, extreme temperatures, salinity, exposure to toxic compounds, inadequate or extreme radiation, plant infection with pathogens, and pest outbreaks [8,9]. Screening plants for various biotic and abiotic stress conditions is vital in breeding and selecting elite varieties. Most plant screening trials for stresses are conducted under field conditions, yet very challenging to manage, both physically and economically, and subject to various risks due to dynamic external environments.

Plant tissue culture provides an effective, efficient, and comparatively economical platform to screen plants for biotic and abiotic stresses. Plant cell and tissue culture, also known as in vitro culture, is based on the cell theory of Schwann and Schleiden (1838) and the ideas of Gottlieb Haberlandt at the beginning of the 20th century [10]. In vitro plant tissue culture is based on cells’ “totipotency” or “total potential”. Theoretically, every cell can become a fully grown plant when provided with suitable conditions. Totipotency has been better described as the ability of any fully functional components of plants to undergo dedifferentiation and redifferentiate to form an organized tissue, structure, and eventually a whole organism [11,12]. Based on this phenomenon, whole plants develop when plant cells or tissues are provided with specific nutrients and optimal growth conditions under an in vitro sterile environment. In vitro culture of plants under defined culture media composition with the ability to impose variables with no external environmental influence while maintaining control environment parameters offers the opportunity for more efficient screening for desirable characteristics. This is also applied to test tolerance to selective agents such as toxins and antibiotics. Utilization of in vitro selection can considerably shorten the time and cost of the selection process under selection pressure with minimal environmental interaction. Conducting in vitro screening for stress will not replace but complement field selection resulting in better insight and outcome. Moreover, it can be used as an early evaluation platform to understand and provide direction with justification for the further need for field evaluation. As with any technique or procedure, in vitro screening for stress tolerance has its challenges. The biggest challenge is the requirement for reliable and established tissue culture protocols for specific plant species. Another issue is the lack of correlation between the mechanisms of tolerance operating at the cellular or tissue level in cultured cells to those of whole plants. Epigenetic adaptation can also interfere with the results as non-tolerant cells may have an epigenetic adaptation during in vitro culture process [13,14,15]. This type of epigenetic adaptation can be overcome using a short-term or one-step in vitro selection process.

Studies on plant biodiversity, especially crop species, have increased tremendously over the years in search of better and more resilient crops despite breeding attempts. The research focused on plant improvement and selection has taken a massive hype for searching elite species for new and better chemicals, disease resistance, productivity, and consumer preference. This review elaborates on how in vitro culture is utilized to screen plant species for different biotic and abiotic stress evaluation studies with examples of a broad variety of crop species. The focus is on in vitro screening for the main stresses: drought, salinity, and disease resistance. This review also highlights other uses of in vitro screening for the identification of metal accumulators and stress-tolerant transgenic plant development with further discussion on the pros and cons of specific studies and the effectiveness of the in vitro culture tools utilized as fast and cost-effective alternative in plant screening.

## 2. In Vitro Screening of Drought Tolerance

Drought affects plant growth and development, reflecting plants’ productivity [16,17]. Drought stress impacts crop performance at several phases in the plant’s life cycle, from emergence to maturity, including seed germination, vegetative growth, and reproductive development, ultimately affecting the quality and quantity of the harvest. Drought, especially in arid and semi-arid regions, causes significant agricultural losses [18]. Under drought stress, several molecular, biochemical, physiological, morphological, and ecological characteristics and processes of plants are affected due to the triggering of stress-responsive factors [19]. As a result, the productivity and quality of plants diminish in water-deficient situations. Growth stages, age, plant species, drought intensity, and duration of drought period are the primary determinants for the responses elicited by the plant in response to drought stimuli.

Plants process various mechanisms to adopt, tolerate, or resist drought conditions. However, the ability and the level of tolerance/resistance differ among and within plant species, especially when genetic variability exists due to outcrossing or natural mutation [16,19,20,21]. Drought responses are governed by activating signal transduction pathways linked with molecular networks to elicit survival or adaptation mechanisms [20]. In general, drought causes a reduction in soil moisture and results in reduced water potential in root cells [17]. For many decades, breeding and selection for drought tolerance or resistance have been major research areas for many crop species i.e., rice, maize, and sorghum [22,23,24,25,26,27]. However, screening for drought in field conditions requires a substantial amount of resources (land, labor, and energy), which is costly, and is associated with challenges in relying on nature for stable environmental conditions to efficiently and effectively replicate data in expressing exact genotype [21].

In vitro applications for drought screening can be a smart and easy method compared to field studies. The strategy to apply drought conditions in vitro is to impose similar conditions created at cellular levels when plants are subjected to drought in the field environment. Under abiotic stress, plants accumulate solutes or osmolytes within the cell due to less water availability. Therefore, high molecular weight solutes such as sucrose, sorbitol, mannitol, and poly-ethylene glycol (PEG) are suitable candidates to impose physiological drought under in vitro conditions [28,29,30,31,32]. In addition, these osmolytes stabilize proteins and cell membranes’ structure during dehydration stress conditions [33,34].

Contrary to drought stress, which induces osmotic stress in plants, accumulating these chemicals reduces osmotic potential, preserving cellular turgor and enhancing water absorption [20,35]. Moreover, they play a crucial function in protecting plant cells from oxidative stress by removing reactive oxygen species [20,32,35]. It has been shown that sucrose accumulates in plant tissues under drought stress [36,37,38]. PEG, sucrose, mannitol, and sorbitol have been the main chemicals for imposing osmotic pressure in vitro. PEG has reportedly been used to impose physiological drought in plants [28,29,39,40]. This high molecular weight chemical is an inert, non-penetrating osmoticum that decreases the water potential of nutritional solutions without being taken up by the plant or phytotoxic. Since PEG does not reach the apoplast, it drives water from the cell wall and interior. Therefore, PEG solutions resemble dry soil more closely than low molecular weight chemicals, which permeate the cell wall with solute. PEG is not used in the cellular metabolism of plants, but it does induce water stress by lowering the water potential of nutrient solutions, hence inhibiting plant development in vitro [28,39,41]. It has no harmful or toxic effects on the plant; nonetheless, it restricts plant development by lowering the water potential of the culture medium, such as water deficit soil, preventing cultured explants from absorbing water [42]. Mannitol and sorbitol have been usually used as osmotic pressure regulators in plant in vitro cultures while utilized as a carbon source [43]. Several studies have applied PEG, mannitol, and sorbitol for in vitro drought screening studies (Table 1).

## 3. In Vitro Screening of Salinity Tolerance

Salinity in soil and water is the most critical constraint that affects plant growth and development [58,59]. Due to osmotic or ionic stress or nutritional imbalance, salinity stress has a deleterious effect on plant development [60,61,62]. Arid and semi-arid environments are characterized by accumulating large quantities of salts in the soil [63]. Although many remedial and management procedures are utilized to make salt-affected soils suitable for agriculture, they are exceedingly costly and do not offer lasting answers to the salinity problem. Therefore, salinity stress has gained considerable traction over the past few decades due to the vast experimental evidence from what has occurred in nature regarding the evolution of highly salt-tolerant ecotypes of various plant species [64,65,66], as well as the remarkable progress made in improving various agronomic traits through artificial selection [67]. Plant tissue culture is the most efficient method for enhancing and producing salt tolerance in plants. By utilizing plant cell and tissue culture, it is possible to focus on the physiological and biochemical processes crucial to the cell and contribute to the alterations brought about by salt stress. Using two in vitro culture methods, salt-tolerant plants have been obtained through cell and tissue culture procedures. The first method involves selecting mutant cell lines from cultivated cells, followed by plant regeneration using these cells (somaclones). The second method is the in vitro screening of plant germplasm for salt tolerance, which has been successfully used in durum wheat [68]. Doubled haploid lines generated from pollen culture of salt-tolerant F1 hybrid parents have the potential to enhance salt tolerance [69,70]. Somaclonal variation and in vitro-induced mutagenesis can create variability from which crop plants can be improved. Examples of other in vitro selections for increased resistance to salt stresses are shown in Table 2. Enhancing resistance to both hyper-osmotic stress and ion toxicity may also be accomplished by molecular breeding of salt-tolerant plants employing molecular markers or genetic engineering.

## 4. In Vitro Screening of Disease Resistance

Plant diseases cause substantial revenue loss in agriculture due to lost or poor performing plants when infected. Thus, breeding and selection for disease resistance are at the forefront of crop science. Various air, soil, and water-borne fungal, bacterial, viral, and mycoplasma diseases affect commercial crops, especially in monoculture and chemically fertilized environments. Therefore, studying and screening for disease tolerance and resistance are routine operations. Often such investigations require specialized conditions and highly controlled setups to minimize the risk of an unintentional spread of diseases to the outside environment causing disease outbreaks for major agricultural crops. In vitro selection using pathogenesis-related proteins, antifungal peptides, or phytoalexin production can help select elite-resistant varieties. This method is simpler and cheaper than generating plants through transgenic technology, which is costly, time-consuming, and more challenging to commercialize due to policy and social acceptance barriers. Exposing organogenic or embryogenic calli, shoots, somatic embryos, or cell suspensions to pathogen toxins, culture filtrate, or the direct pathogen can effectively screen plant samples for pathogen resistance in vitro. In Table 3, such in vitro screening studies are listed for various crop species.

Some research suggests that rather than the success of screening, somaclonal variation occurring during the tissue culture process is a probable factor in disease-resistant behavoir [91,92,93,94]. A combination of a chance mutation in vitro with in vitro selection pressure appears to delay and confound the later examination of plants produced by these methods. In such an attempt by Vos et al. (2000), they grew tens of thousands of standard seedlings in culture and screened in vitro for resistance to guava wilt disease [95]. This is the most impressive example of such in vitro screening study for disease resistance. This accelerated the discovery of potentially resistant plants, saving the South African guava industry.

## 5. In Vitro Screening of Metal Hyperaccumulators

Phytoremediation is a novel and cost-effective method for removing hazardous heavy metals (Pb, Cd, Cu, Zn, etc.) and organic contaminants from water and soil [7]. There are now accessible biotechnologies for better comprehending plants’ mechanism of heavy metal absorption and examining their potential for remediation enhancement [96]. On the other hand, metal accumulators are highly beneficial and trendy as food supplements or for recouping rare and expensive metal elements for cosmetics and other uses [97]. When studying the tolerance of plant cells to hazardous substances, in vitro cultures provide several advantages [98]. In this regard, in vitro screening is a preliminary technique for assessing woody plant materials since it reduces the time required for growth and treatment and the amount of space necessary for the tests. Since it is conducted under controlled conditions, plant tissue culture is one of the most dependable procedures used in fundamental research to establish the metabolic capacity of plants [99]. Research on phytoremediation typically uses several plant tissue cultures as model plant systems. Some examples of these cultures are calli, cell suspensions, and hairy roots. When it comes to research on the inherent metabolic capacities of plant cells and their ability to tolerate toxicity, in vitro cultures provide several benefits to the otherwise unavailable experimentation process. In the quest for fundamental information about plants, the capacity to determine the specific contributions that plant cells make to the process of pollutant absorption and detoxification in the absence of interference by microbes is of special value. However, the final objective of such studies is to develop a realistic phytoremediation technology. In that case, it is necessary to understand the inherent limitations in using in vitro cultures as a representative of entire plants in the field. It is highly likely that the bioavailability of contaminants and the processes of pollutant uptake and metabolite distribution will be significantly different in the two systems. This can lead to qualitative and quantitative differences in metabolic profiles and tolerance characteristics. In order to gain complete understanding or to identify an effective species in phytoremediation through chemical accumulation, it is necessary to use intact rooted plantlets in tissue culture conditions for screening studies. However, several studies have shown that plant tissue culture is a handy tool in the field of phytoremediation surveys (Table 4). The findings obtained from tissue cultures may be utilized to make predictions regarding the reactions of plants to environmental toxins, as well as to enhance the design of future traditional whole plant tests, which in turn helps to lower their overall costs.

## 6. Stress Tolerance through Transgenic Technology

Genetic modification technology for agriculturally important plant species has achieved major advances in the last decade. The development of transgenic plants with desirable characteristics, such as tolerance to biotic and abiotic stress, is a reality. Plant characteristics are altered much faster than ever by utilizing a wide variety of approaches through gene transfer and gene editing [2,4,117]. The technique for tissue regeneration through tissue culture is a prerequisite in such processes, and it is vital.

Genetic transformation has been proposed for several decades as a quick way to modify the morphological characteristics of an organism. Plant genetic transformation techniques can be classified as direct and vector-based, introducing transgenic DNA to the host organism. Direct genetic transformation refers to the direct introduction of transgenic DNA to a plant cell. The most used techniques are biolistics and biological vectors that use *Agrobacterium tumefaciens*-mediated transformation [2].

Genetic transformation through biolistics can transform any totipotent plant cell, from which cell lines, tissues, or whole plants can be created. However, it has the disadvantage that the transformation can be transient and generate chimeric plants (non-transformed cells within the plant), in addition to the requirement of expensive equipment and low transformation efficiency [118]. On the other hand, the most studied and used vector-based transformation method is through infection of *Agrobacterium tumefaciens*, a natural plant pathogenic bacterium capable of incorporating a DNA region (Ti plasmid of the *Agrobacterium tumefaciens*) into the plant genome. This system does not require specialized equipment and thus is inexpensive, and the number of transformation events per cell is limited. However, limitations exist due to plant regeneration challenges from the transformed callus cells [119,120,121].

The methods of introducing foreign DNA or changing plant genomes have been updated for the use of more defined transformation systems such as the “Clustered Regularly Interspaced Short Palindromic Repeats (CRISPR) -associated protein 9 (Cas9) systems”. This system is powerful to allow specific genetic edits to change genomes according to the need. The CRISPR/Cas9 system is based on the immune system of bacteria adapted to eukaryotic systems, including plants [122,123], whose principle is the RNA–DNA interaction to search for the sequence genomics [124,125].

**Figure 1:**
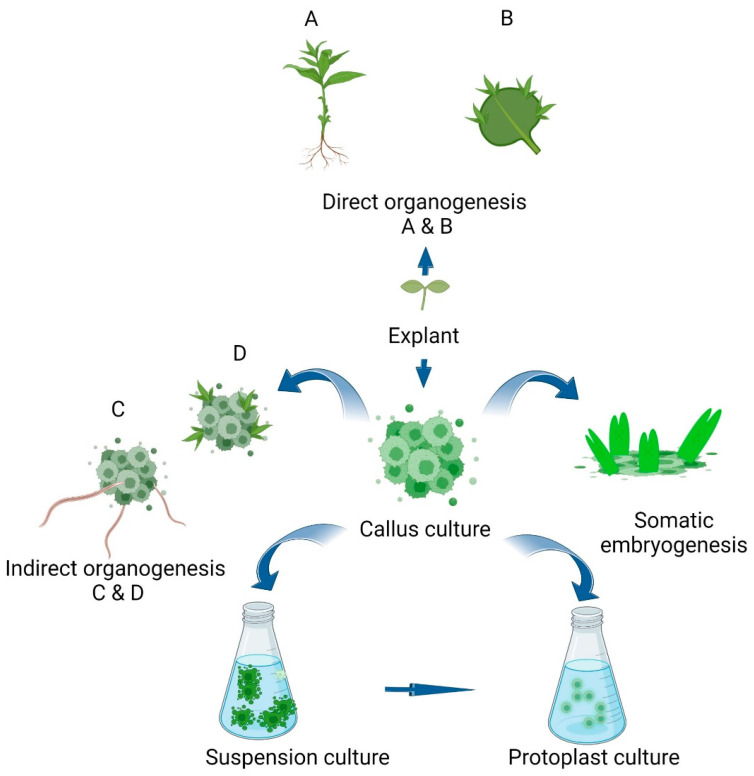
Different techniques in tissue culture for plant regeneration can be utilized for the selection and genetic transformation of plants. Starting from explants under selection mediums, direct organogenesis can be achieved (A and B) or indirect organogenesis (C and D) through an intermediate callus phase. Further, callus can be used to form intact plantlets through an embryonic pathway or in suspension culture directly or via protoplast culture techniques in genetic transformation attempts.

Not all plant tissue types and species are conducive to the above transformation methods. An explant can be a variety of tissues, depending on the particular plant species and its regenerative ability. Table 5 below lists several studies conducted to achieve abiotic and biotic resistance through genetic transformation. Identification of a suitable tissue culture approach to maximize the transformation efficiency with higher regeneration of transformed cells is critical. Different approaches are illustrated in Figure 1.

Different combinations of culture type and transformation protocol are used depending on the plant species and cultivar. In some species, various culture types and regeneration methods can be used, which enables a wide variety of transformation protocols to be utilized. However, there is no choice over culture type and regeneration method in other species, limiting the applicable transformation protocols [136].

## 7. Summary

Salinity, drought, water logging, heat, frost, and mineral toxicities limit commercial agricultural productivity. Biotechnology can bring in solutions to increase crop productivity. Tissue culture-based in vitro selection and mutagenesis have become a viable and affordable method for stress-tolerant plant development. Current research supports the notion that in vitro screening is an alternative and a support platform for stress tolerance screening for drought, salinity, and chemical toxicity. Further, in vitro culture permits accurate modification and assessment of stress variables, identifying stress-tolerance genes and metabolic pathways. Expanding research into this area of science will ensure more efficient and effective screening methodologies for the future of sustainable agriculture.

## Figures and Tables

**Table 1:** Application of different chemicals of in vitro screening for drought tolerance.

Plant Species	Marial Screened under In Vitro Conditions	In Vitro Screening Method	Reference
*Soybean * (*Glycine max*) *Cultivars: B 3731, MLG 2999, MSC 8606,Tidar*	Immature cotyledons were used as explant/Somatic embryogenesis	Subjected to 15% PEG 6000. The application of PEG was terminated after the plants were 28 days old.	[39]
*Soybean * (*Glycine max*) *Cultivars:* JS335, JS9305	Calli/cell clumps/embryoids rose from the immature and mature embryonic axis and cotyledons	Subjected to discontinuous exposure to a lethal dose of 20% PEG6000	[40]
Durum wheat (*Triticum durum*) *Cultivars:* Waha, Oued Zenati, Djenah Khetifa	Immature embryo-derived calli	Subjected to 10% and 20% PEG 6000	[44]
Durum wheat (*Triticum durum Desf.*) *Cultivars:* Karim, Sebou, Ourigh, Anouar	Immature embryo-derived calli	Subjected to 10% and 20% PEG 10000	[45]
Sorghum (*Sorghum bicolor* L. *Moench*)	Embryogenic callus	Subjected to a range of 0–15% PEG 8000	[46]
Wheat (*Triticum aestivum*) *Cultivar:* GA-2002	Immature embryo-derived calli	Subjected to a range of PEG 6000	[47]
Wheat (*Triticum aestivum*) *Cultivars:* Sakha 8, Sakha 69, Giza 157, Sids 1, West bred, Falke, Hahn/Turaco, and Kauz/Gen	Immature embryo-derived calli	Subjected to a range of Mannitol	[48]
Tagetes (*Tagetes minuta*)	Calli-derived from cotyledon explants	Subjected to a range of Mannitol	[49]
Sweet leaf (*Stevia rebaudiana*)	Nodal shoot/micro propagated shoots	Subjected to a range of PEG 6000	[50]
Potato (*Solanum tuberosum*) 27 CIP different cultivars	Nodal cuttings	Subjected to a range of Sorbitol	[51]
Rice (*Oryza sativa*) *Cultivars: PA U 201 and PR 116*	Embryogenic calli	Subjected to a range of (0–2%) PEG 6000	[52]
Potato (*Solanum tuberosum*) *Cultivars:* IWA-1, IWA-3, IWA-5	Well-sprouted microtubers	Subjected to a range of Sorbitol and PEG 6000	[30]
Rice (*Oryza sativa*) *Cultivars: IR 18351-229-3, IR 3185-6-3-3-2, SR 26-B, Nona Bokra, and C 14-8*	Seed-derived calli	Subjected to a range of PEG 6000	[53]
Ground nut (*Arachis hypogaea*) *Cultivars: TMV2, JL24*	Hypocotyl-derived calli	Subjected to a range of PEG (0.0, 0.4, 0.6, 0.8 and 1.0 MPa)	[54]
Brown mustard (*Brassica juncea Czern*) *Cultivars: RW-85-59*	Cotyledon-derived calli	Subjected to a range of Mannitol	[55]
Coconut (*Cocos nucifera*) *Variety: Sri Lanka tall*	Embryo cultures	Subjected to a range of PEG 6000 (1–5%)	[56]
Sugarcane (*Saccharum* sp.) *Cultivars: R570 and CP59-73*	Calli cultures	Subjected to a range of Mannitol	[57]

**Table 2:** In vitro selection for increased resistance to salt stresses.

Plant Species	Marial Screened under In Vitro Conditions	In Vitro Screening/ Mutagenesis Method	Reference
*Limnophila aromatica* (*Lamk.*) *Merr.*	In vitro organogenesis from nodal explant	Nodal explants subjected to callus formation on a series of NaCl concentrations 0–100 mM	[71]
*Bacopa monnieri* (L.) *Wettst.*	In vitro organogenesis from nodal explant	Nodal explants subjected to callus formation on a series of NaCl concentrations 0–100 mM	[71]
Aubergine (*Solanum melongena*) *Cultivar: Bonica*	Leaf segment-derived calli	Callus formation on a series of NaCl concentrations 40–120 mM	[72]
Sweet potato (*Ipomoea batatas*) Cultivar: Shiroyutaka	Embryogenic calli	Callus formation on a series of NaCl concentrations 25–200 mM	[73]
Potato (*Solanum tuberosum*) Cultivars: Kennebec, Norchip, Red Pontiac, Russet Burbank, Russet Norkotah, and Superior	Stem cuttings, Leaf rachis originated callus-derived cell cultures	Callus formation on a series of NaCl concentrations 0.25–0.5 M	[74]
Canola (*Brassica napus*) Cultivars: Bingo Torpe, Conny and Siberian.	Hypocotyls and Cotyledonary-derived calli	Callus formation on a series of NaCl concentrations 4000, 8000, 12,000, and 16,000 ppm	[75]
Tomato (*Solanum lycopersicom*) Cultivars: Nora, PS-10, Peto, Roma	Hypocotyl-derived calli	Callus formation on a series of NaCl concentrations 5, 50, 75, and 100 mM	[76]
Chrysanthemum (*Chrysanthemum morifolium Ramat.*) Cultivar: Maghi Yellow	Ray floret-derived calli	Callus formation on a series of NaCl concentrations 50, 75 and 100 mM	[77]
Sour Orange (*Citrus aurantium* L.)	Embryogenic calli	Callus formation on a series of NaCl concentrations 100–300 mM	[78]
Sugarcane (*Saccharum sp.*) Cultivar: CP65-357	Young leaf-derived callus	Callus formation on a 68 mM NaCl concentration	[79]
Bamboo (*Dendrocalamus strictus Nees*)	Embryogenic calli	Callus formation on a series of NaCl concentrations 50, 100, 150, 200, and 250 mM	[80]
Carrot (*Daucus carota subsp. sativus* L.) Cultivars: Dolanka and two Iranian landraces (DAL and NL)	Protoplasts	Protoplast culture in a series of NaCl concentrations 10–400 mM	[81]
Rice (*Oryza sativa* L.) Cultivars: KDML and LPT	Embryogenic calli	Callus formation on a series of NaCl concentrations 1–2%	[82]
Durum wheat (*Triticum turgidum* var. *durum*)	Immature embryogenic calli	Callus formation on a series of NaCl concentrations 0.3, 0.6, 0.9, 1.2, 1.5, 1.8, and 2.1% *w*/*v*	[68]

**Table 3:** In vitro selection for increased resistance to biotic stresses.

Plant Species	Marial Screened under In Vitro Conditions	In Vitro Screening/Investigation Method	Reference
*Lycoris radiata*	Callus induced from meristem tissue of dried bulbs	Wounding stress imposed to analyse the accumulation of galantamine content as a measure of abiotic stress response in plants	[83]
Ground nut (*Arachis hypogaea*) *Cultivars: VRI-2, TMV-7*	Immature leaf-derived calli	Culture filtrate of *Cercosporidium personation*	[84]
*Femminello’ lemon* (*Citrus* *limon* L.) *Burro. f.* *Tarocco’ orange* (*Citrus sinensis* L. *Osb.*)	Nucellar calli	Culture filtrate and toxin of *Phoma tracheiphila*	[85]
Turmeric (*Curcuma longa*) *Cultivar: Suguna*	Non-embryogenic propagule-derived calli	Culture filtrate of *Pythium graminicolum*	[86]
Cotton (*Gossypium hirsutum* L.) Cultivar: SVPR 2	Hypercotyl-derived somatic embryos	Culture filtrate of *Fusarium oxysporum* and *Alternaria macrospora*	[87]
Sugarcane (*Saccharum sp.*) Cultivars: CoJ 88 and CoJ 64	Apical spindle tips-derived calli	Culture filtrate of *Colletotrichum falcatum*	[88]
Wheat (*Triticum aestivum*) Varieties: Sumai 3 (P1), Mianyang 11 (P2), and their reciprocal F1 hybrids	Embryo-derived calli	Deoxynivalenol	[89]
grapevine (*Vitis vinifera* L.) *Cultivar: Chardonnay*	Proembryogenic masses derived calli	Culture filtrate of *Elsinoe ampelina*	[90]
*Populus nigra × trichocarpa*	Internode-derived callus, Somaclonal selection	Culture filtrate of *Septoria musiva*	[91]
Sweet orange (*Citrus sinensis Osbeck*)	embryogenic callus mutagenesis with EMS by somaclones tolerant	Culture filtrate of *Xanthomonas citri subsp. citri*	[92]
Peach (*Prunus persica*) Cultivars: Sunhigh, Redhaven	Embryo-derived calli	Culture filtrate of *Xanthomonas campestris pv. pruni*	[93]

**Table 4:** In vitro selection for increased resistance to hyperaccumulators.

Plant Species	Tissue Culture Method	Stress	Reference
Potato (*Solanum tuberosum* L.) Cultivar: Iwa	Micropropagation in vitro cell line selection	Cadmium	[100]
Foxtail millet (*Setaria Italica*)	Leaf base and mesocotyl explant-derived calli	Zinc	[101]
Rice (*Oryza sativa* L.)	Embryo-derived calli	Aluminium	[102,103,104]
*Brassica campestris cv. M27* *Brassica juncea cv. Pusabold*	Cotyledon-derived calli	Zinc	[105]
Jungle rice (*Echinochloa colona*)	Leaf base-derived calli	Chromium, Nickel	[106]
Tobacco (*Nicotiana tabacum* L.) *Cultivar: Xanthi*	Leaf-derived calli	Copper	[107]
Mustard (*Brassica juncea*)	Hypocotyl-derived calli	Cadmium	[108,109]
Silver poplar (*Populus alba*)	Microshoot cultures	Cadmium, Copper	[110]
*Prunella vulgaris*	Shoot-tip explants	Cadmium, Zinc	[111]
Downy *oak* (*Quercus pubescens*)	Seedlings	Cadmium, Copper	[112]
Silver poplar (*Populus alba*)	Microshoots culture	Arsenic, Copper, Cadmium, Zinc	[113]
Flax (*Linum usitatissimum* L.)	Cell culture/Calli/shoot-tip culture	Cadmium	[114,115,116]

**Table 5:** Examples of attempts to improve abiotic stress tolerance in crop plants through genetic transformation.

Crop	Trait	Trasformation Tissue Type	Reference
Apple (*Malus domestica Borkh.*) Cultivar Royal Gala.	Herbicide resistance	Callus/Organogenesis	[126]
Apple (*Malus domestica*) Cultivar: Mailing 26	Resistance to Erwínía amylovora	Callus/Organogenesis	[127]
Apricot (*Prunus armeniaca*) Cultivar: Kecskemeter	Plum pox virus resistance	Callus/Organogenesis	[128]
European plum (*Prunus domestica*) Cultivar: Stanley	Papaya ringspot virus resistance	Callus/Organogenesis	[129,130]
Maize (*Zea mays*)	Salt resistance	Embryo culture/Organogenesis	[131]
Rice (*Oryza sativa*)	Salt resistance	Callus/Organogenesis	[132]
Chickpea (*Cicer arietinum*) Cultivar: C 235 (desi type)	Drought resistance	Callus/Direct organogenesis	[133]
Colt cherry (*Prunus avium × pseudocerasus*)	Salt and drought resistance	Protoplast	[134]
Pistachio (*Pistacia vera*) Cultivar: Sarakhs	Drought resistance	Somatic embryogenesis	[135]

## Data Availability

Not applicable.

## References

[1] Kumar P.P., Loh C.S. (2012). Plant Tissue Culture for Biotechnology. Plant Biotechnology and Agriculture.

[2] Wijerathna-Yapa A., Ramtekey V., Ranawaka B., Basnet B.R. (2022). Applications of in Vitro Tissue Culture Technologies in Breeding and Genetic Improvement of Wheat. Plants.

[3] Gamborg O.L. (2002). Plant Tissue Culture. Biotechnology. Milestones. Vitr. Cell Dev. Biol. Plant.

[4] Wijerathna-Yapa A., Pathirana R. (2022). Sustainable Agro-Food Systems for Addressing Climate Change and Food Security. Agriculture.

[5] Tripathi A.D., Mishra R., Maurya K.K., Singh R.B., Wilson D.W. (2019). Estimates for World Population and Global Food Availability for Global Health. The Role of Functional Food Security in Global Health.

[6] Valoppi F., Agustin M., Abik F., Morais de Carvalho D., Sithole J., Bhattarai M., Varis J.J., Arzami A.N., Pulkkinen E., Mikkonen K.S. (2021). Insight on Current Advances in Food Science and Technology for Feeding the World Population. Front. Sustain. Food Syst..

[7] Choudhary R., Hiti-Bandaralage J.C.A., Ahlawat J., Gaur N., Diwan B., Iqbal H.M.N., Bilal M., Nguyen T.A. (2022). 13—Nanobioremediation: An Introduction. Nano-Bioremediation: Fundamentals and Applications.

[8] Pessarakli M. (2019). Handbook of Plant and Crop Stress.

[9] Fahad S., Nie L., Chen Y., Wu C., Xiong D., Saud S., Hongyan L., Cui K., Huang J. (2015). Crop Plant Hormones and Environmental Stress. Sustainable Agriculture Review.

[10] Thorpe T.A. (2007). History of Plant Tissue Culture. Mol. Biotechnol..

[11] Su Y.H., Tang L.P., Zhao X.Y., Zhang X.S. (2021). Plant Cell Totipotency: Insights into Cellular Reprogramming. J. Integr. Plant Biol..

[12] Fehér A. (2019). Callus, Dedifferentiation, Totipotency, Somatic Embryogenesis: What These Terms Mean in the Era of Molecular Plant Biology?. Front. Plant Sci..

[13] Smỳkal P., Valledor L., Rodriguez R., Griga M. (2007). Assessment of Genetic and Epigenetic Stability in Long-Term in Vitro Shoot Culture of Pea (*Pisum Sativum* L.). Plant Cell Rep..

[14] Smulders M.J.M., De Klerk G.J. (2011). Epigenetics in Plant Tissue Culture. Plant Growth Regul..

[15] Miguel C., Marum L. (2011). An Epigenetic View of Plant Cells Cultured in Vitro: Somaclonal Variation and Beyond. J. Exp. Bot..

[16] Fahad S., Bajwa A.A., Nazir U., Anjum S.A., Farooq A., Zohaib A., Sadia S., Nasim W., Adkins S., Saud S. (2017). Crop Production under Drought and Heat Stress: Plant Responses and Management Options. Front. Plant Sci..

[17] Gupta A., Rico-Medina A., Caño-Delgado A.I. (2020). The Physiology of Plant Responses to Drought. Science.

[18] Leng G., Hall J. (2019). Crop Yield Sensitivity of Global Major Agricultural Countries to Droughts and the Projected Changes in the Future. Sci. Total Environ..

[19] Sehgal A., Sita K., Siddique K.H.M., Kumar R., Bhogireddy S., Varshney R.K., HanumanthaRao B., Nair R.M., Prasad P.V.V., Nayyar H. (2018). Drought or/and Heat-Stress Effects on Seed Filling in Food Crops: Impacts on Functional Biochemistry, Seed Yields, and Nutritional Quality. Front. Plant Sci..

[20] Seleiman M.F., Al-Suhaibani N., Ali N., Akmal M., Alotaibi M., Refay Y., Dindaroglu T., Abdul-Wajid H.H., Battaglia M.L. (2021). Drought Stress Impacts on Plants and Different Approaches to Alleviate Its Adverse Effects. Plants.

[21] Hoekstra F.A., Golovina E.A., Buitink J. (2001). Mechanisms of Plant Desiccation Tolerance. Trends Plant Sci..

[22] Cattivelli L., Rizza F., Badeck F.-W., Mazzucotelli E., Mastrangelo A.M., Francia E., Marè C., Tondelli A., Stanca A.M. (2008). Drought Tolerance Improvement in Crop Plants: An Integrated View from Breeding to Genomics. Field Crops Res..

[23] Badu-Apraku B., Obisesan O., Abiodun A., Obeng-Bio E. (2021). Genetic Gains from Selection for Drought Tolerance during Three Breeding Periods in Extra-Early Maturing Maize Hybrids under Drought and Rainfed Environments. Agronomy.

[24] Nuccio M.L., Paul M., Bate N.J., Cohn J., Cutler S.R. (2018). Where Are the Drought Tolerant Crops? An Assessment of More than Two Decades of Plant Biotechnology Effort in Crop Improvement. Plant Sci..

[25] Kumar A., Dixit S., Ram T., Yadaw R.B., Mishra K.K., Mandal N.P. (2014). Breeding High-Yielding Drought-Tolerant Rice: Genetic Variations and Conventional and Molecular Approaches. J. Exp. Bot..

[26] Hu H., Xiong L. (2014). Genetic Engineering and Breeding of Drought-Resistant Crops. Annu. Rev. Plant Biol..

[27] Yahaya M.A., Shimelis H. (2022). Drought Stress in Sorghum: Mitigation Strategies, Breeding Methods and Technologies—A Review. J. Agron. Crop Sci..

[28] Mehmandar M.N., Rasouli F., Giglou M.T., Zahedi S.M., Aazami M.A. (2021). In Vitro Screening of *Cucmis Melo* L. Against Drought Mediated by PEG and Sorbitol. Res. Squre.

[29] Abdelrahem A., Ragab R., Ahmed K., Omar, Dakhly F., Mohamed S. In Vitro Selection for Tomato Plants for Drought Tolerance via Callus Culture under Polyethylene Glycol (PEG) and Mannitol Treatments. Proceedings of the 8th African Crop Science Society Conference.

[30] Gopal J., Iwama K. (2007). In Vitro Screening of Potato against Water-Stress Mediated through Sorbitol and Polyethylene Glycol. Plant Cell Rep..

[31] Samarina L., Matskiv A., Simonyan T., Koninskaya N., Malyarovskaya V., Gvasaliya M., Malyukova L., Tsaturyan G., Mytdyeva A., Martinez-Montero M.E. (2020). Biochemical and Genetic Responses of Tea (*Camellia Sinensis* (L.) Kuntze) Microplants under Mannitol-Induced Osmotic Stress In Vitro. Plants.

[32] Darko E., Végh B., Khalil R., Marček T., Szalai G., Pál M., Janda T. (2019). Metabolic Responses of Wheat Seedlings to Osmotic Stress Induced by Various Osmolytes under Iso-Osmotic Conditions. PLoS ONE.

[33] Gekko K., Timasheff S.N. (1981). Mechanism of Protein Stabilization by Glycerol: Preferential Hydration in Glycerol-Water Mixtures. Biochemistry.

[34] Wingsle G., Karpinski S., Hällgren J.E. (1999). Low Temperature, High Light Stress and Antioxidant Defence Mechanisms in Higher Plants. PHYTON-HORN-.

[35] Yang X., Lu M., Wang Y., Wang Y., Liu Z., Chen S. (2021). Response Mechanism of Plants to Drought Stress. Horticulturae.

[36] Hoffmann C. (2010). Sucrose Accumulation in Sugar Beet Under Drought Stress. J. Agron. Crop Sci..

[37] Taji T., Ohsumi C., Iuchi S., Seki M., Kasuga M., Kobayashi M., Yamaguchi-Shinozaki K., Shinozaki K. (2002). Important Roles of Drought- and Cold-Inducible Genes for Galactinol Synthase in Stress Tolerance in Arabidopsis Thaliana. Plant J..

[38] Lu J., Sun M., Ma Q., Kang H., Liu Y., Hao Y., You C. (2019). MdSWEET17, a Sugar Transporter in Apple, Enhances Drought Tolerance in Tomato. J. Integr. Agric..

[39] Sunaryo W., Widoretno W., Nurhasanah N., Sudarsono S. (2016). Drought Tolerance Selection of Soybean Lines Generated from Somatic Embryogenesis Using Osmotic Stress Simulation of Polyethylene Glycol (PEG). Nusant. Biosci..

[40] Mishra N., Tripathi M.K., Tiwari S., Tripathi N., Sapre S., Ahuja A., Tiwari S. (2021). Cell Suspension Culture and In Vitro Screening for Drought Tolerance in Soybean Using Poly-Ethylene Glycol. Plants.

[41] Muscolo A., Sidari M., Anastasi U., Santonoceto C., Maggio A. (2014). Effect of PEG-Induced Drought Stress on Seed Germination of Four Lentil Genotypes. J. Plant Interact..

[42] Bressan R.A., Hasegawa P.M., Handa A.K. (1981). Resistance of Cultured Higher Plant Cells to Polyethylene Glycol-Induced Water Stress. Plant Sci. Lett..

[43] de Paiva Neto V.B., Otoni W.C. (2003). Carbon Sources and Their Osmotic Potential in Plant Tissue Culture: Does It Matter?. Sci. Hortic..

[44] Kacem N.S., Delporte F., Muhovski Y., Djekoun A., Watillon B. (2017). In Vitro Screening of Durum Wheat against Water-Stress Mediated through Polyethylene Glycol. J. Genet. Eng. Biotechnol..

[45] Bouiamrine E.H., Diouri M. (2012). Response of Durum Wheat (*Triticum Durum* Desf.) Callus Culture to Osmosis- Induced Drought Stress Caused by Polyethylene Glycol (PEG). Ann. Biol. Res..

[46] Duncan R.R., Waskom R.M., Nabors M.W. (1995). In Vitro Screening and Field Evaluation of Tissue-Culture-Regenerated Sorghum (*Sorghum Bicolor* (L.) Moench) for Soil Stress Tolerance. Euphytica.

[47] Mahmood I., Razzaq A., Ashraf M., Hafiz I.A., Kaleem S., Qayyum A., Ahmad M. (2012). In Vitro Selection of Tissue Culture Induced Somaclonal Variants of Wheat for Drought Tolerance. J. Agric. Res..

[48] Abdel-Ghany H.M., Nawar A.A., Ibrahim M.E., El-Shamarka A., Selim M.M., Fahmi A.I. Using Tissue Culture to Select for Drought Tolerance in Bread Wheat. Proceedings of the 4th International Crop Science Congress.

[49] Mohamed M.A., Harris P.J., Henderson J. (2000). In Vitro Selection and Characterisation of a Drought Tolerant Clone of Tagetes Minuta. Plant Sci..

[50] Ahmad M.A., Javed R., Adeel M., Rizwan M., Yang Y. (2020). PEG 6000-Stimulated Drought Stress Improves the Attributes of In Vitro Growth, Steviol Glycosides Production, and Antioxidant Activities in Stevia Rebaudiana Bertoni. Plants.

[51] Gelmesa D., Dechassa N., Mohammed W., Gebre E., Monneveux P., Bündig C., Winkelmann T. (2017). In Vitro Screening of Potato Genotypes for Osmotic Stress Tolerance. Open Agric..

[52] Wani S., Sofi P., Gosal S., Singh N. (2010). In Vitro Screening of Rice (*Oryza Sativa* L) Callus for Drought Tolerance. Commun. Biometry Crop Sci..

[53] Biswas J., Chowdhury B., Bhattacharya A., Mandal A.B. (2002). In Vitro Screening for Increased Drought Tolerance in Rice. Vitr. Cell Dev. Biol. Plant.

[54] Purushotham M.G., Patil V., Chandrashekra Raddey P., Prasad T.G., Vajranabhaiah S.N. (1998). Development of in Vitro PEG Stress Tolerant Cell Lines in Two Groundnut (*Arachis Hypogaea* L.) Genotypes. Indian J. Plant Physiol..

[55] Gangopadhyay G., Basu S., Gupta S. (1997). In Vitro Selection and Physiological Characterization of NaCl- and Mannitol-Adapted Callus Lines in Brassica Juncea. Plant Cell Tissue Organ Cult..

[56] Karunaratne S., Santha S., Kovoor A. (1991). An in Vitro Assay for Drought-Tolerant Coconut Germplasm. Euphytica.

[57] Errabii T., Gandonou C.B., Essalmani H., Abrini J., Idaomar M., Skali-Senhaji N. (2006). Growth, Proline and Ion Accumulation in Sugarcane Callus Cultures under Drought-Induced Osmotic Stress and Its Subsequent Relief. Afr. J. Biotechnol..

[58] Parihar P., Singh S., Singh R., Singh V.P., Prasad S.M. (2015). Effect of Salinity Stress on Plants and Its Tolerance Strategies: A Review. Environ. Sci. Pollut. Res..

[59] Zhao C., Zhang H., Song C., Zhu J.-K., Shabala S. (2020). Mechanisms of Plant Responses and Adaptation to Soil Salinity. Innovation.

[60] Munns R., Tester M. (2008). Mechanisms of Salinity Tolerance. Annu. Rev. Plant Biol..

[61] Fang S., Hou X., Liang X. (2021). Response Mechanisms of Plants Under Saline-Alkali Stress. Front. Plant Sci..

[62] Rao Y., Peng T., Xue S. (2023). Mechanisms of Plant Saline-Alkaline Tolerance. J. Plant Physiol..

[63] EL Sabagh A., Hossain A., Barutçular C., Iqbal M.A., Islam M.S., Fahad S., Sytar O., Çiğ F., Meena R.S., Erman M., Fahad S., Hasanuzzaman M., Alam M., Ullah H., Saeed M., Ali Khan I., Adnan M. (2020). Consequences of Salinity Stress on the Quality of Crops and Its Mitigation Strategies for Sustainable Crop Production: An Outlook of Arid and Semi-Arid Regions. Environment, Climate, Plant and Vegetation Growth.

[64] Munns R., Day D.A., Fricke W., Watt M., Arsova B., Barkla B.J., Bose J., Byrt C.S., Chen Z.-H., Foster K.J. (2020). Energy Costs of Salt Tolerance in Crop Plants. New Phytol..

[65] Isayenkov S.V. (2019). Genetic Sources for the Development of Salt Tolerance in Crops. Plant Growth Regul..

[66] Basu S., Kumar A., Benazir I., Kumar G. (2021). Reassessing the Role of Ion Homeostasis for Improving Salinity Tolerance in Crop Plants. Physiol. Plant..

[67] Ashraf M., Wu L. (1994). Breeding for Salinity Tolerance in Plants. Crit. Rev. Plant Sci..

[68] Arzani A., Mirodjagh S.-S. (1999). Response of Durum Wheat Cultivars to Immature Embryo Culture, Callus Induction and in Vitro Salt Stress. Plant Cell Tissue Organ Cult..

[69] Senadhira D., Zapata-Arias F.J., Gregorio G.B., Alejar M.S., de la Cruz H.C., Padolina T.F., Galvez A.M. (2002). Development of the First Salt-Tolerant Rice Cultivar through Indica/Indica Anther Culture. Field Crops Res..

[70] Kenny L., Caligari P.D.S. (1996). Androgenesis of the Salt Tolerant Shrub Atriplex Glauca. Plant Cell Rep..

[71] Dogan M. (2020). Effect of Salt Stress on in Vitro Organogenesis from Nodal Explant of *Limnophila Aromatica* (Lamk.) Merr. And *Bacopa Monnieri* (L.) Wettst. And Their Physio-Morphological and Biochemical Responses. Physiol. Mol. Biol. Plants.

[72] Hannachi S., Werbrouck S., Bahrini I., Abdelgadir A., Siddiqui H.A., Van Labeke M.C. (2021). Obtaining Salt Stress-Tolerant Eggplant Somaclonal Variants from In Vitro Selection. Plants.

[73] Anwar N., Kikuchi A., Watanabe K.N. (2010). Assessment of Somaclonal Variation for Salinity Tolerance in Sweet Potato Regenerated Plants. Afr. J. Biotechnol..

[74] Naik P.S., Widholm J.M. (1993). Comparison of Tissue Culture and Whole Plant Responses to Salinity in Potato. Plant Cell Tiss. Organ Cult..

[75] Abdrabou R., Fergani M.A., Azzam C.R., Morsi N. (2017). Devolopment of Some Canola Genotypes to Salinity Tolerance Using Tissue Culture Technique. Egypt. J. Agron..

[76] Aazami M.A., Rasouli F., Ebrahimzadeh A. (2021). Oxidative Damage, Antioxidant Mechanism and Gene Expression in Tomato Responding to Salinity Stress under in Vitro Conditions and Application of Iron and Zinc Oxide Nanoparticles on Callus Induction and Plant Regeneration. BMC Plant Biol..

[77] Hossain Z., Mandal A.K.A., Datta S.K., Biswas A.K. (2007). Development of NaCl-Tolerant Line in Chrysanthemum Morifolium Ramat. Through Shoot Organogenesis of Selected Callus Line. J. Biotechnol..

[78] Koc N.K., Bas B., Koc M., Kusek M. (2009). Investigations of in Vitro Selection for Salt Tolerant Lines in Sour Orange (*Citrus Aurantium* L.). Biotechnology.

[79] Gandonou C.B., Errabii T., Abrini J., Idaomar M., Senhaji N.S. (2006). Selection of Callus Cultures of Sugarcane (*Saccharum* Sp.) Tolerant to NaCl and Their Response to Salt Stress. Plant Cell Tiss. Organ Cult..

[80] Singh M., Jaiswal U., Jaiswal V.S. (2003). In Vitro Selection of NaCl-Tolerant Callus Lines and Regeneration of Plantlets in a Bamboo (*Dendrocalamus Strictus* Nees.). Vitr. Cell Dev. Biol. Plant.

[81] Kiełkowska A., Grzebelus E., Lis-Krzyścin A., Maćkowska K. (2019). Application of the Salt Stress to the Protoplast Cultures of the Carrot (*Daucus Carota* L.) and Evaluation of the Response of Regenerants to Soil Salinity. Plant Cell Tiss. Organ Cult..

[82] Vajrabhaya M., Thanapaisal T., Vajrabhaya T. (1989). Development of Salt Tolerant Lines of KDML and LPT Rice Cultivars through Tissue Culture. Plant Cell Rep..

[83] Park C.H., Sathasivam R., Nguyen B.V., Baek S.-A., Yeo H.J., Park Y.E., Kim H.H., Kim J.K., Park S.U. (2020). Metabolic Profiling of Primary Metabolites and Galantamine Biosynthesis in Wounded Lycoris Radiata Callus. Plants.

[84] Venkatachalam P., Jayabalam N. (1997). Selection and Regeneration of Groundnut Plants Resistant to the Pathotoxic Culture Filtrate OfCercosporidium Personation through Tissue Culture Technology. Appl. Biochem. Biotechnol..

[85] Gentile A., Tribulato E., Continella G., Vardi A. (1992). Differential Responses of Citrus Calli and Protoplasts to Culture Filtrate and Toxin of Phoma Tracheiphila. Theoret. Appl. Genet..

[86] Gayatri M.C., Darshini V.R., Kavyashree R. (2005). Selection of Turmeric Callus Tolerant to Culture Filtrate of Pythium Graminicolum and Regeneration of Plants. Plant Cell Tiss. Organ Cult..

[87] Ganesan M., Jayabalan N. (2006). Isolation of Disease-Tolerant Cotton (*Gossypium Hirsutum* L. Cv. SVPR 2) Plants by Screening Somatic Embryos with Fungal Culture Filtrate. Plant Cell Tiss. Organ Cult..

[88] Sengar A.S., Thind K.S., Kumar B., Pallavi M., Gosal S.S. (2009). In Vitro Selection at Cellular Level for Red Rot Resistance in Sugarcane (*Saccharum* Sp.). Plant Growth Regul..

[89] Yang Z., Yang X., Huang D. (1998). Studies on Somaclonal Variants for Resistance to Scab in Bread Wheat (*Triticum Aestivum* L.) through in Vitro Selection for Tolerance to Deoxynivalenol. Euphytica.

[90] Jayasankar S., Li Z., Gray D.J. (2000). In-Vitro Selection of Vitis Vinifera `Chardonnay’ with Elsinoe Ampelina Culture Filtrate is Accompanied by Fungal Resistance and Enhanced Secretion of Chitinase. Planta.

[91] Ostry M.E., Ward K.T. (2003). Field Performance of Populus Expressing Somaclonal Variation in Resistance to Septoria Musiva. Plant Sci..

[92] Ge H., Li Y., Fu H., Long G., Luo L., Li R., Deng Z. (2015). Production of Sweet Orange Somaclones Tolerant to Citrus Canker Disease by in Vitro Mutagenesis with EMS. Plant Cell Tiss. Organ Cult..

[93] Hammerschlag F.A., Ognjanov V. (1990). Somaclonal Variation in Peach: Screening for Resistance to Xanthomonas Campestris Pv. Pruni and Pseudomonas Syringae Pv. Syringae. Acta Hortic..

[94] Larkin P.J., Scowcroft W.R. (1981). Somaclonal Variation—A Novel Source of Variability from Cell Cultures for Plant Improvement. Theoret. Appl. Genet..

[95] Vos J.E., Schoeman M.H., Berjak P., Watt M.P., Toerien A.J. In Vitro Selection and Commercial Release of Guava Wilt Resistant Rootstocks. Proceedings of the XXV International Horticultural Congress, Part 3: Culture Techniques with Special Emphasis on Environmental Implications 513.

[96] Capuana M. (2011). Heavy Metals and Woody Plants-Biotechnologies for Phytoremediation. Iforest-Biogeosci. For..

[97] O’Donohue B., Hiti-Bandaralage J., Gleeson M., O’Brien C., Harvey M.-A., van der Ent A., Pinto Irish K., Mitter N., Hayward A. (2022). Tissue Culture Tools for Selenium Hyperaccumulator Neptunia Amplexicaulis for Development in Phytoextraction. Nat. Prod. Bioprospect..

[98] Golan-Goldhirsh A., Barazani O., Nepovim A., Soudek P., Smrcek S., Dufkova L., Krenkova S., Yrjala K., Schröder P., Vanek T. (2004). Plant Response to Heavy Metals and Organic Pollutants in Cell Culture and at Whole Plant Level. J. Soils Sediments.

[99] Confalonieri M., Balestrazzi A., Bisoffi S., Carbonera D. (2003). In Vitro Culture and Genetic Engineering of *Populus* spp.: Synergy for Forest Tree Improvement. Plant Cell Tissue Organ Cult..

[100] Ashrafzadeh S., Leung D.W.M. (2017). Novel Potato Plants with Enhanced Cadmium Resistance and Antioxidative Defence Generated after in Vitro Cell Line Selection. PLoS ONE.

[101] Samantaray S., Rout G.R., Das P. (1999). In Vitro Selection and Regeneration of Zinc Tolerant Calli from *Setaria italica* L. Plant Sci..

[102] Bertin P., Bouharmont J. (1997). Use of Somaclonal Variation and in Vitro Selection for Chilling Tolerance Improvement in Rice. Euphytica.

[103] Roy B., Mandal A.B. (2005). Towards Development of Al-Toxicity Tolerant Lines in Indica Rice by Exploiting Somaclonal Variation. Euphytica.

[104] Van Sint Jan V., Costa de Macedo C., Kinet J.-M., Bouharmont J. (1997). Selection of Al-Resistant Plants from a Sensitive Rice Cultivar, Using Somaclonal Variation, in Vitro and Hydroponic Cultures. Euphytica.

[105] Rout G.R., Samantaray S., Das P. (1999). In Vitro Selection and Biochemical Characterisation of Zinc and Manganese Adapted Callus Lines in *Brassica* Spp. Plant Sci..

[106] Samantaray S., Rout G.R., Das P. (2001). Induction, Selection and Characterization of Cr and Ni-Tolerant Cell Lines of *Echinochloa Colona* (L.) Link in Vitro. J. Plant Physiol..

[107] Rout G.R., Sahoo S. (2007). In Vitro Selection and Plant Regeneration of Copper-Tolerant Plants from Leaf Explants of Nicotiana *Tabacum* L. Cv. ‘Xanthi’. Plant Breed..

[108] Gasic K., Korban S.S. (2007). Expression of Arabidopsis Phytochelatin Synthase in Indian Mustard (*Brassica Juncea*) Plants Enhances Tolerance for Cd and Zn. Planta.

[109] Liang Zhu Y., Pilon-Smits E.A., Jouanin L., Terry N. (1999). Overexpression of Glutathione Synthetase in Indian Mustard Enhances Cadmium Accumulation and Tolerance. Plant Physiol..

[110] Marzilli M., Di Santo P., Palumbo G., Maiuro L., Paura B., Tognetti R., Cocozza C. (2018). Cd and Cu Accumulation, Translocation and Tolerance in *Populus Alba* Clone (Villafranca) in Autotrophic in Vitro Screening. Environ. Sci. Pollut. Res..

[111] Çetin B., Ay Ç. (2020). Determination of CD (İİ) and ZN (İİ) Tolerance and Phytoremediation Ability of *Prunella Vulgaris* L. Plant Tissue Culture Conditions.

[112] Di Santo P., Cocozza C., Tognetti R., Palumbo G., Iorio E.D., Paura B. (2016). A Quick Screening to Assess the Phytoextraction Potential of Cadmium and Copper in Quercus Pubescens Plantlets. iForest-Biogeosci. For..

[113] Di Lonardo S., Capuana M., Arnetoli M., Gabbrielli R., Gonnelli C. (2011). Exploring the Metal Phytoremediation Potential of Three *Populus Alba* L. Clones Using an in Vitro Screening. Environ. Sci. Pollut. Res..

[114] Berková V., Berka M., Griga M., Kopecká R., Prokopová M., Luklová M., Horáček J., Smýkalová I., Čičmanec P., Novák J. (2022). Molecular Mechanisms Underlying Flax (*Linum Usitatissimum* L.) Tolerance to Cadmium: A Case Study of Proteome and Metabolome of Four Different Flax Genotypes. Plants.

[115] Hradilová J., Řehulka P., Řehulková H., Vrbová M., Griga M., Brzobohatý B. (2010). Comparative Analysis of Proteomic Changes in Contrasting Flax Cultivars upon Cadmium Exposure. Electrophoresis.

[116] Smykalova I., Vrbova M., Tejklova E., Vetrovcova M., Griga M. (2010). Large Scale Screening of Heavy Metal Tolerance in Flax/Linseed (*Linum Usitatissimum* L.) Tested in Vitro. Ind. Crops Prod..

[117] Bowerman A.F., Byrt C.S., Roy S.J., Whitney S.M., Mortimer J.C., Ankeny R.A., Gilliham M., Zhang D., Millar A.A., Rebetzke G.J. (2023). Potential Abiotic Stress Targets for Modern Genetic Manipulation. Plant Cell.

[118] Sanford J.C. (1990). Biolistic Plant Transformation. Physiol. Plant..

[119] Hiei Y., Ishida Y., Komari T. (2014). Progress of Cereal Transformation Technology Mediated by Agrobacterium Tumefaciens. Front. Plant Sci..

[120] Niazian M., Sadat Noori S.A., Galuszka P., Mortazavian S.M.M. (2017). Tissue Culture-Based Agrobacterium-Mediated and in Planta Transformation Methods. Czech J. Genet. Plant Breed..

[121] Paes de Melo B., Lourenço-Tessutti I.T., Morgante C.V., Santos N.C., Pinheiro L.B., de Jesus Lins C.B., Silva M.C.M., Macedo L.L.P., Fontes E.P.B., Grossi-de-Sa M.F. (2020). Soybean Embryonic Axis Transformation: Combining Biolistic and Agrobacterium-Mediated Protocols to Overcome Typical Complications of In Vitro Plant Regeneration. Front. Plant Sci..

[122] Xing H.-L., Dong L., Wang Z.-P., Zhang H.-Y., Han C.-Y., Liu B., Wang X.-C., Chen Q.-J. (2014). A CRISPR/Cas9 Toolkit for Multiplex Genome Editing in Plants. BMC Plant Biol..

[123] Svitashev S., Schwartz C., Lenderts B., Young J.K., Mark Cigan A. (2016). Genome Editing in Maize Directed by CRISPR–Cas9 Ribonucleoprotein Complexes. Nat. Commun..

[124] Arora L., Narula A. (2017). Gene Editing and Crop Improvement Using CRISPR-Cas9 System. Front. Plant Sci..

[125] Wada N., Ueta R., Osakabe Y., Osakabe K. (2020). Precision Genome Editing in Plants: State-of-the-Art in CRISPR/Cas9-Based Genome Engineering. BMC Plant Biol..

[126] Yao J.-L., Cohen D., Atkinson R., Richardson K., Morris B. (1995). Regeneration of Transgenic Plants from the Commercial Apple Cultivar Royal Gala. Plant Cell Rep..

[127] Norelli J.L., Aldwinckle H.S., Destéfano-Beltrán L., Jaynes J.M. (1994). Transgenic ‘Mailing 26′Apple Expressing the Attacin E Gene Has Increased Resistance to Erwinia Amylovora. Euphytica.

[128] da Câmara Machado M.L., da Câmara Machado A., Hanzer V., Weiss H., Regner F., Steinkellner H., Mattanovich D., Plail R., Knapp E., Kalthoff B. (1992). Regeneration of Transgenic Plants of Prunus Armeniaca Containing the Coat Protein Gene of Plum Pox Virus. Plant Cell Rep..

[129] Scorza R., Levy L., Damsteegt V., Yepes L.M., Cordts J., Hadidi A., Slightom J., Gonsalves D. (1995). Transformation of Plum with the Papaya Ringspot Virus Coat Protein Gene and Reaction of Transgenic Plants to Plum Pox Virus. J. Am. Soc. Hortic. Sci..

[130] Scorza R., Ravelonandro M., Callahan A.M., Cordts J.M., Fuchs M., Dunez J., Gonsalves D. (1994). Transgenic Plums (*Prunus Domestica* L.) Express the Plum Pox Virus Coat Protein Gene. Plant Cell Rep..

[131] Zhang M., Cao Y., Wang Z., Wang Z., Shi J., Liang X., Song W., Chen Q., Lai J., Jiang C. (2018). A Retrotransposon in an HKT1 Family Sodium Transporter Causes Variation of Leaf Na+ Exclusion and Salt Tolerance in Maize. New Phytol..

[132] Santosh Kumar V.V., Verma R.K., Yadav S.K., Yadav P., Watts A., Rao M.V., Chinnusamy V. (2020). CRISPR-Cas9 Mediated Genome Editing of Drought and Salt Tolerance (OsDST) Gene in Indica Mega Rice Cultivar MTU1010. Physiol. Mol. Biol. Plants.

[133] Bhatnagar-Mathur P., Vadez V., Jyostna Devi M., Lavanya M., Vani G., Sharma K.K. (2009). Genetic Engineering of Chickpea (*Cicer Arietinum* L.) with the P5CSF129A Gene for Osmoregulation with Implications on Drought Tolerance. Mol. Breed..

[134] Ochatt S.J., Power J.B. (1989). Selection for Salt and Drought Tolerance in Protoplast- and Explant-Derived Tissue Cultures of Colt Cherry (*Prunus Avium* × *Pseudocerasus*). Tree Physiol..

[135] Ghadirzadeh-Khorzoghi E., Jahanbakhshian-Davaran Z., Seyedi S.M. (2019). Direct Somatic Embryogenesis of Drought Resistance Pistachio (*Pistacia Vera* L.) and Expression Analysis of Somatic Embryogenesis-Related Genes. S. Afr. J. Bot..

[136] Walden R., Wingender R. (1995). Gene-transfer and plant-regeneration (techniques). Trends Biotechnol..

